# Closing-wedge distal femoral osteotomy combined with medial patellofemoral ligament reconstruction for recurrent patellar dislocation with genu valgum

**DOI:** 10.1186/s12891-021-04554-5

**Published:** 2021-08-09

**Authors:** Lizhong Jing, Xiaole Wang, Xiaoliang Qu, Kun Liu, Xiaotan Wang, Lu Jiang, Di Wu, Zhiwei Zhang, Zhuang Li, Le Yu, Shaoshan Wang, Jiushan Yang

**Affiliations:** 1 Department of Orthopedics, Affiliated Hospital to Shandong University of Traditional Chinese Medicine, Jinan, 250011 China; 2 Department of Orthopedics, Dongying Hospital of Traditional Chinese Medicine, Dongying, 257000 China; 3 Department of Orthopedics, Penglai Traditional Chinese Medicine Hospital, Yantai, 265600 China; 4 Shandong University of Traditional Chinese Medicine, Jinan, 250000 China

**Keywords:** Closing-wedge distal femoral osteotomy, Medial patellofemoral ligament reconstruction, Recurrent patellar dislocation, Genu valgum

## Abstract

**Background:**

Medial patellofemoral ligament reconstruction (MPFLR) is a well-established procedure for addressing recurrent patellar dislocation (RPD) in young patients. However, despite being a promising procedure for RPD with genu valgum, there is a scarcity of reports on simultaneous MPFLR and closing-wedge distal femoral osteotomy (CWDFO). The purpose of the present study was to observe and analyse the clinical and imaging findings of CWDFO combined with MPFLR for RPD with genu valgum.

**Methods:**

From May 2015 to April 2018, 25 patients with RPD and genu valgum were surgically treated in our department. Anteroposterior long-leg, weight-bearing, lower-extremity radiographs, lateral radiographs and computed tomography (CT) scans of the patellofemoral joint were obtained, and the anatomical femorotibial angle (aFTA), mechanical lateral distal femoral angle (mLDFA), weight-bearing line rate (WBLR), patellar height, patellar lateral shift (PLS) and tibial tubercle–trochlear groove (TT-TG) distance were analysed. Validated knee scores, such as the Kujala, Lysholm, visual analogue scale (VAS) scores and Tegner socres, were evaluated preoperatively and 2 years postoperatively.

**Results:**

25 patients, with an average age of 19.8 years (14–27), were evaluated. During the 2-year follow-up period, all patients were able to achieve a better sports level without any problems, with no recurrence of patellar instability. Compared with preoperation, the aFTA, mLDFA, WBLR and PLS showed statistically significant improvement following the procedure (p < 0.001). Meanwhile, no significant differences in the Insall index and TT-TG distance were found. The mean Kujala score, average Lysholm score, VAS score and Tegner socres showed significant postoperative improvement.

**Conclusions:**

CWDFO combined with MPFLR is a suitable treatment for RPD with genu valgum, and can lead to significant improvement in the clinical and imaging findings of the knee in the short term.

## Background

RPD is relatively common among teenagers [1], with numerous factors contributing thereto, such as patella alta, femoral malrotation, tibial tubercle lateralisation, and genu valgum [2, 3] Thus, there is need for an individual treatment option based on potential pathoanatomy.

Genu valgum has been proven to considerably influence patellar tracking [4]. For patients with genu valgum, applying medialisation of the tibial tuberosity (TTM) may not be appropriate, since the contact pressure between the patellofemoral and the medial tibiofemoral is noticeably increased, thereby changing the balance of tibiofemoral joint loading [5]. MPFLR is the most common surgical procedure for PRD, but genu valgum is a significant risk factor that can lead to failure of an isolated MPFLR [6, 7]. Further, the risk of degeneration in the lateral femorotibial compartment has been found to be 2 times greater in 3-degree malalignment of the valgus [8, 9]. Hence, as a significant risk factor for RPD, correcting the valgus deformity simultaneously could be a reasonable approach.

In general, both patellar instability and maltracking need to be corrected for RPD [8, 9]. In terms of patellar instability, the widely accepted treatment option is MPFLR [10]. However, as patellar maltracking could be restored by DFO in patients with genu valgum [11] CWDFO combined with MPFLR could be justified in these patients.

However, there is currently a scarcity of studies examining the clinical and imaging findings of the treatment of RPD due to valgus deformity by DFO combined with MPFLR. In addition, up to now, there has been no study with a relatively larger number of patients. The purpose of the present study was to analyse the short-term results of CWDFO combined with MPFLR for RPD with genu valgum. An assumption was made that excellent clinical and imaging results could be obtained through this procedure in the patients after a follow-up period of at least 2 years. To the present knowledge, among all related studies currently published, the present study has the largest number of patients.

## Methods

### Study design

The indication for surgical treatment was RPD (≥2 dislocations) of the knee combined with genu valgum deformity. The exclusion criteria were acute lateral patellar dislocation, habitual patellar dislocation, and any combination thereof with patella alta or femoral intorsion.

The present study was given approval by the Ethics Committee of our hospital and informed consent was signed by each patient.

There was no control group.

### Clinical and radiological evaluation

All patients were evaluated preoperatively and 2 years postoperatively according to the Kujala, Lysholm, visual analogue scale (VAS) scores and Tegner scores. The preoperative and 2-year postoperative radiographic examinations of the knee included anteroposterior (AP) long-leg, weight-bearing, lower-extremity radiographs and lateral radiographs to assess the anatomical femorotibial angle (aFTA), mechanical lateral distal femoral angle (mLDFA), weight-bearing line rate (WBLR) and patellar height. The patellar height was measured using the method applied by Insall [12]. The patellar lateral shift (PLS) was calculated by CT at 30 degrees of knee flexion, as described by Nha et al. [11], and was defined as the shortest distance from the cortex of the lateral trochlea to the lateral edge of the patella. The profile information of these patients is presented in Table 1.

**Table 1:** Profile information of the study groups

	CWDFO, MPFLR	CWDFO, MPFLR, LR
Total knees	14	11
Age (years)	18 (14-27)	20 (15-26)
Male	6	7
Female	8	4
Preoperative aFTA (°)	163.43±3.84	166.01±4.59
Correction angle	10.17±2.13	8.75±1.71
Postoperative aFTA (°)	174.10±3.04	176.38±2.55
TT-TG (mm)	16.33±2.16	19.07±3.05
TT medialization (mm)	---	---
Insall index	1.03±0.24	1.09±0.16
Follow-up duration (months)	35(24-57)	37(27-51)

*CWDFO* closing-wedge distal femoral osteotomy, *LR* lateral release, *aFTA* anatomical femorotibial angle, *MPFLR* medial patellofemoral ligament reconstruction, *TT-TG* tibial tubercle–trochlear groove, *TT* tibial tuberosity

### Surgical technique

An arthroscopy was firstly performed to remove any loose bodies, which confirmed patellar maltracking.

**Fig. 1:**
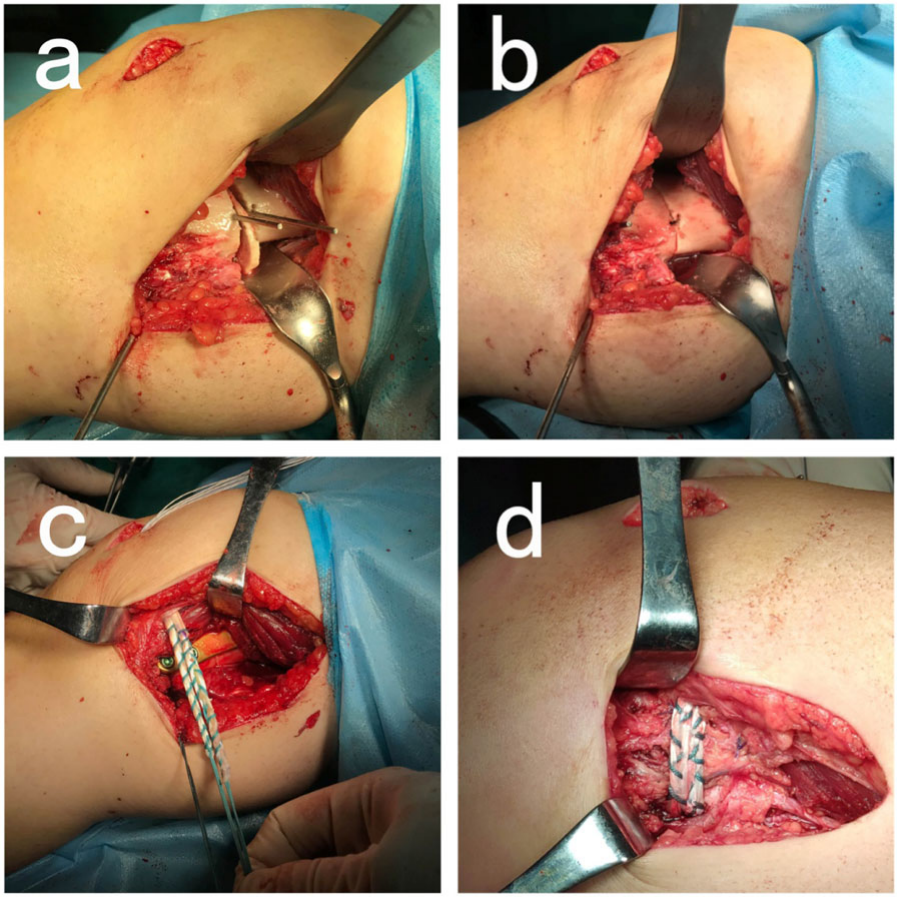
Intraoperative setting of the CWDFO. Removing the wedge (**a**). Closing the medial osteotomy carefully (**b**). Performing osteosynthesis with a plate, and fixing a semitendinosus graft in the patella and anatomical femoral insertion site (**c**, **d**)

For CWDFO, preoperative planning followed the method described by Paley et al. [13]. The target line in the present study passed 50% of the plateau width from the inside margin to that of the outside. The operative manipulation was conducted according to the method presented by Sabbag OD et al. [14]. A Hohmann retractor was used to protect the dorsal and ventral soft tissue, while the TomoFix plate was used to stabilise the osteotomy area (Fig. 1 a, b).

MPFLR was performed on all patients according to Schoettle’s technique [15]. For the majority of cases, the entrance of the femoral tunnel was posterior to the far end of the plate, and thus, the distant unicortical locking screws were replaced by shorter ones if the tunnel was violated thereby. Following plate fixation and femoral tunnel preparation, the vastus medialis fascia was closed covering the plate. The autologous semitendinosus graft was fixed in the medial margin of patella by two absorbable suture anchors, passed subcutaneously and superficial to the vastus medialis fascia over the plate, and fixed by an unabsorbable interference screw in the anatomical femoral insertion site (Fig. 1 c, d).

Subsequently, patellar tracking was re-evaluated. If there was obvious patellar tilt and a tight tension of lateral soft tissue, arthroscopic lateral release with a thermal device was then performed.

### Rehabilitation

A standardised rehabilitation protocol was executed. On the second day postoperation, passive knee flexion, isometric quadriceps contraction and ankle pumps were performed according to the prescription. After 4 weeks, partial weight-bearing exercise was allowed. For the first 6 weeks after surgery, patients were instructed to perform passive flexion step-by-step to a full range of movement, and were required to wear a knee brace. After 8 weeks, all patients were instructed to gradually return to full weight-bearing. After 4 months, participation in non- competitive sports was allowed. The patients could gradually return to competitive sports as tolerated after 6 months.

### Statistical analysis

The preoperative and postoperative results for all cases were compared using paired tests. Variables were compared on the basis of independent sample tests, and included the Lysholm scores, Kujala scores, VAS scores, Tegner scores, aFTA, mLDFA, WBLR, Insall index, PLS and TT-TG. The average data of said measurements were used in the analysis process. In the present study, SPSS (version 24, SPSS, Inc., Chicago, Illinois) was used to perform statistical analysis. All reported *P* values were 2-tailed, and *P* values less than 0.05 were considered to indicate statistical significance.

## Results

**Fig. 2:**
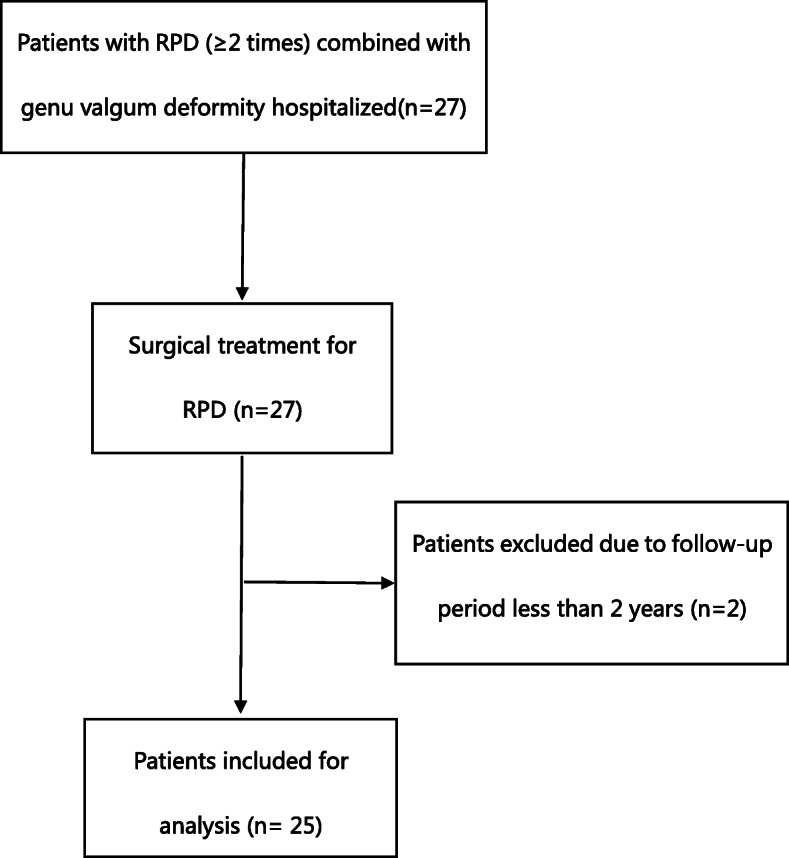
flowchart of patients selection

From May 2015 to April 2018, 27 patients underwent CWDFO combined with MPFLR at our institution. Among the patients, 2 patients were unwilling to further participate in the clinical follow-up period after 1 year postoperatively. Accordingly, 25 of 27 patients (92.5%, 9 women, 16 men) were available for more than 2 years of clinical follow-up observation (Fig. 2), with an average age of 19.8 years (14–27) and an average follow-up duration of 36.67 months (24–57). Right-sided patellar instability was present in 15 patients (60%), while instability on the left side was present in 10 patients (40%). All patients returned to non-competitive sporting activities without any problems, and there has been no reoccurrence of RPD.

**Fig. 3:**
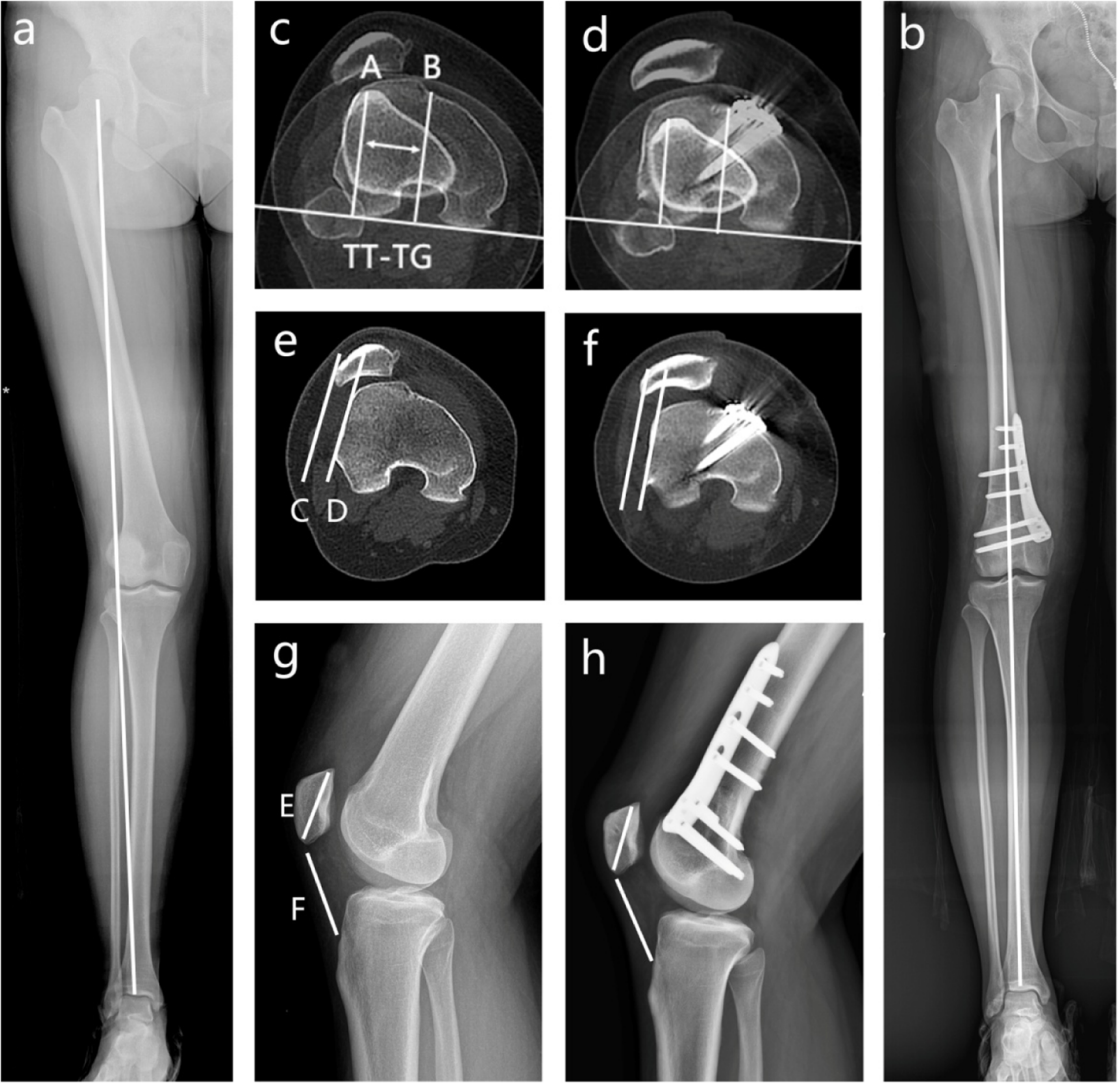
A 19-year-old girl with RPD and valgus deformity of the lower limb. The alignment of the leg axis, TT-TG distance, PLS and insall index of preoperation (**a**,**c**,**e**,**g**) and postoperation (**b**,**d**,**f**,**h**), respectively

**Fig. 4:**
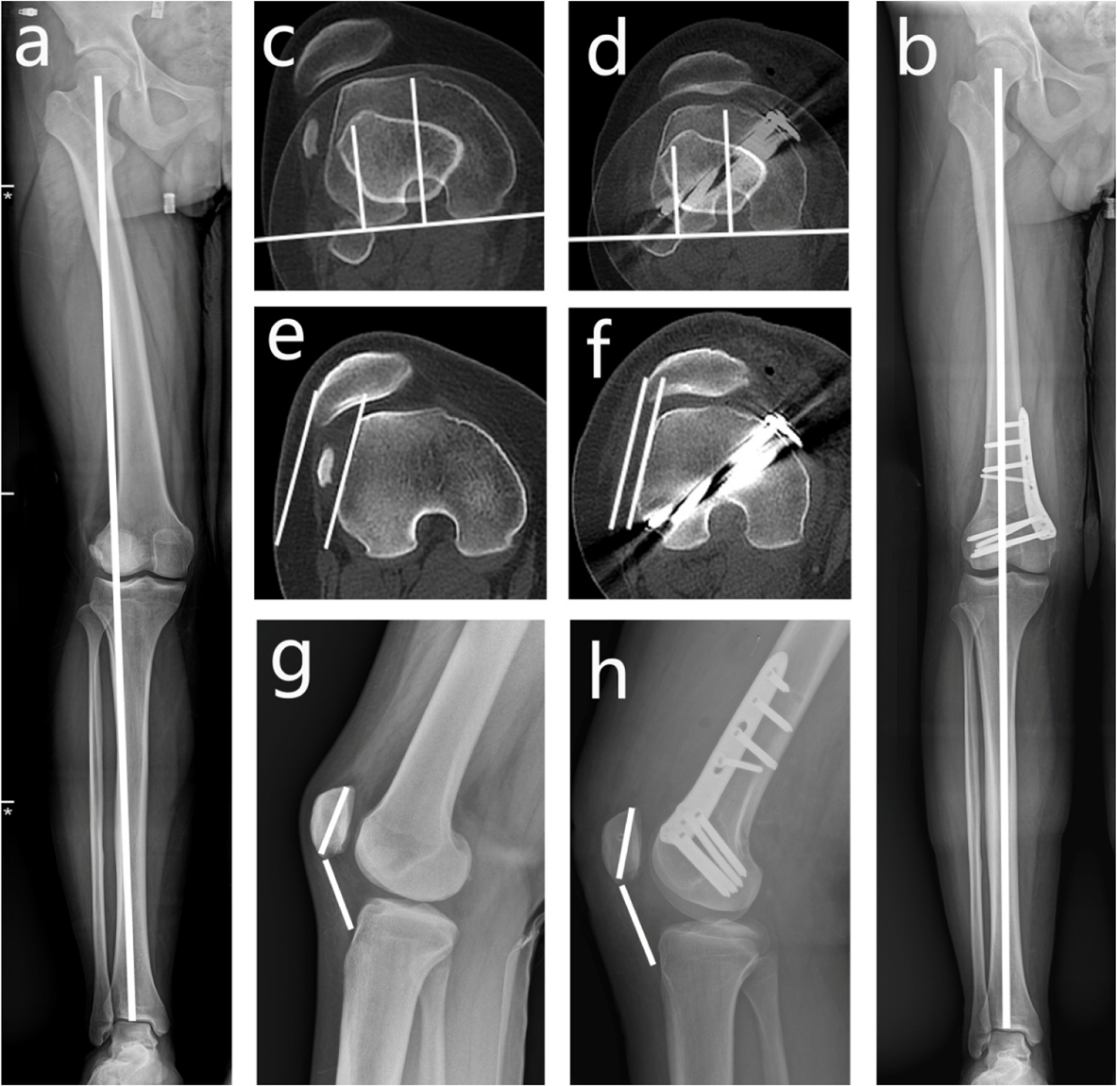
A 16-year-old boy with RPD and valgus deformity of the lower limb. The alignment of the leg axis, TT-TG distance, PLS and insall index of preoperation (**a**,**c**,**e**,**g**) and postoperation (**b**,**d**,**f**,**h**), respectively

Compared with preoperation, following the procedure, the aFTA, WBLR, mLDFA and PLS exhibited statistically significant improvement (*p* < 0.001). Meanwhile, no significant differences in the Insall index and TT-TG distance were found at 2 years postoperation. The results are specified in Table 2, Figs. 3 and 4.

**Table 2:** Preoperative and postoperative radiological parameters

Scale	Preoperatively	2 years postoperatively	*p* value
aFTA (°)	164.67±3.04	174.53±2.42	<0.001
WBLR (%)	74.13±3.68	50.20±2.76	<0.001
mLDFA (°)	81.13±1.68	86.93±1.98	<0.001
Insall	1.04±0.12	1.03±0.11	0.712
PLS (mm)	12.53±2.42	4.60±1.50	<0.001
TT-TG (mm)	17.20±3.84	17.53±3.20	0.739

*WBLR* weight-bearing line rate, *mLDFA* mechanical lateral distal femoral angle, *PLS* patellar lateral shift

The mean Kujala score, average Lysholm score, VAS score and Tegner score are presented in Table 3. All applied knee scores showed statistically significant improvement postoperation.

**Table 3:** Preoperative and postoperative knee scores for patients

Scale	Preoperatively	2 years postoperatively	*p* value
Lysholm	47.87±5.25	84.27±4.11	<0.001
Kujala	48.20±5.57	82.53±4.04	<0.001
VAS	5.07±1.28	1.80±1.2	<0.001
Tegner	2(1-4)	4(3-6)	<0.001

*VAS* visual analogue scale

Complications were found in only one patient, who developed knee stiffness 12 weeks postoperatively. With manipulation under anaesthesia, said patient gradually achieved full range of motion. No patients developed infection or non-union from the surgical procedure. About 3 years after primary surgery, 11 patients had the hardware removed and have had no complications thus far.

## Discussion

The most significant finding in the present study is that the treatment of RPD with genu valgum by CWDFO combined with MPFLR is effective. Through said procedure, radiological correction of the patellofemoral instability and genu valgum could be achieved, and improvement in clinical scores could be obtained.

RPD is connected to various anatomic abnormalities, such as trochlear dysplasia, femoral malrotation, tibial tubercle lateralisation, and genu valgum. As a means to mitigate the countless risk factors for patellar instability, a multitude of methods have been introduced, yet there is still heated debate over which is the optimal procedure.

Although TTM and isolated MPFLR have been well established as effective procedures to treat RPD [16–18], these approaches are not without flaws. With regard to MPFLR, Shah et al. [19] revealed that the overall incidence rate of complications is 26.1%, with common complications including fracture of patellar, residual instability, pain and flexion loss. Among those who underwent MPFLR, up to 38% to 40% of cases reported having anterior knee pain [20]. Additionally, Gobbi et al. [21] suggested that patellar shift and tilt correction will not improve significantly when performing isolated MPFLR in patients with RPD, while patellar maltracking has been regarded as a pathoanatomic risk factor for RPD [22, 23].

Several authors have stated that because of altered contact pressure, osteoarthritis of the patellofemoral and tibiofemoral joints will occur after TTM. In a cadaveric study, Kuroda et al. [5] observed that the contact pressure of the patellofemoral and the medial tibiofemoral compartment significantly increased after TTM. Lobner et al. [24] found that patients treated by TTM suffered with increased damage of retropatellar cartilage and worse pain during activity. In a controlled laboratory study, Mani et al. [25] considered that the pressure applied to tibiofemoral cartilage could be altered by tibiofemoral kinematic changes after TTM.

The normal aFTA, anatomical axes of the femoral and tibial diaphyses is 173–175°. If the aFTA is <173°, then this is deemed genu valgum. In skeletally immature adolescents, a minor valgus deformity of 5–10° is generally considered a physiological issue that usually corrects itself spontaneously with age [26]. However, in skeletally mature patients, this issue is generally considered a pathological issue. Certain kinds of genu valgum deformities require effective correction, including those in patients with progressive genu valgum, or genu valgum combined with the gonarthrosis, such as lateral tibiofemoral compartment osteoarthritis and RPD. Genu valgum is a significant risk factor for failure of an isolated MPFLR [6, 7]. In addition, genu valgum has been regarded as being conducive to increasing the forced lateral displacement of patella and causing a J-sign during the final extension of the knee [9, 27]. MPFL serves to restrict lateral translation of the patella, yet does not pull the patella into the trochlear groove. For this reason, isolated MPFLR should not be recommended in patients with genu valgum [28].

The aforementioned problems could be resolved by DFO in patients with associated genu valgum deformity. Traditionally, DFO was regarded as a surgical option for lateral tibiofemoral compartment osteoarthritis, which could be attributed to genu valgum in many cases [29]. However, in recent years, correction of genu valgum has been generally accepted to increase stabilisation of the patella and reduce the risk of RPD by decreasing the forced shift towards the lateral side applied to the patella [11]. Such findings are of considerable significance because genu valgum is an accepted risk factor for RPD, and also relatively common among teenagers. The procedure for correcting genu valgum is divided into opening-wedge distal femoral osteotomy (OWDFO) and closing-wedge distal femoral osteotomy (CWDFO). Compared with the former, the latter is advantageous in that there is no increase in patellofemoral joint pressure, no need for bone graft and high chance of bone healing [30]. Thus, CWDFO has gained more recognition. For RPD without genu valgum deformity, MPFLR with or without TTM may well suffice [30]. However, the risk of degeneration in the lateral femorotibial compartment has been found to be 2 times greater in 3-degree malalignment of the valgus [8, 9]. Hence, despite CWDFO being major surgery, through a one-stage operation, this procedure could obtain satisfactory results in solving RPD and potential lateral tibiofemoral compartment osteoarthritis [11, 14, 31]. Nha et al .[11] investigated the outcomes of 14 patients who underwent CWDFO over a mean follow-up period of 30 months. The mLDFA changed notably from 83 to 89, while the average distance of the PLS decreased from 13.5 mm to 2.0 mm. The subjective symptoms of all patients were significantly improved after the operation, and there was no RPD. Dickschas [31] studied 18 patients who underwent CWDFO over an average follow-up period of 44 months, and no redislocation was found after surgery. The VAS score for anterior knee pain improved from 5.6 to 2.1.

DFO alone or in combination with different soft tissue procedures for RPD has been reported. The treatment of RPD with CWDFO was first reported in 2009 by Omidi et al. [32], who found that the correction of genu valgum deformity alone can improve the patellofemoral congruence angle and reduce the probability of patellar dislocation. Since then, many studies on this procedure have been reported. Chang et al. [30] examined 10 patients who underwent CWDFO with lateral retinacular release and tightening of the medial retinaculum over an average follow-up period of 20 months. The postoperative KSS and Kujala scores were significantly improved, and patellar dislocation did not recur. Purushothaman et al. [33] reported the case of an RPD patient treated by OWDFO combined with MPFLR, who achieved satisfactory postoperative results at the 1-year follow-up examination. Frings J [23] reported the results of 12 patients with RPD of genu valgus treated with CWDFO combined with MPFLR and TTM. Compared with those before the operation, The VAS score, Kujala score, Lysholm score and Tegner score were significantly improved, and there was no reoccurrence of RPD. In RPD patients with genu valgum deformity, DFO alone only converts complex patellar dislocation to simple patellar dislocation. Without associated soft tissue repair, the disabled MPFL and femoral trochlear abnormalities are still potential risks for RPD. Some authors also deemed it necessary to address all predisposing risk factors, by medial soft-tissue imbrication or reconstruction in addition to realignment osteotomy, to obtain better outcomes [10, 14]. Further, MPFLR has been shown to result in higher functional scores than medial retinacular constriction [34]. Hence, in the present study, CWDFO combined with MPFLR was selected to treat the patients, and the results verified the reliability of the procedure.

McWalter et al. [4] stated that varus or valgus deformities of the leg axis could significantly affect patellar tracking. To be specific, such deformities had a strong impact on the patellar tilt, yet no significant difference was found in the level or slope of lateral translation [4]. However, Nha et al. [11] found that CWDFO could decrease the mean distance of PLS from 13.5 mm to 2.0 mm, which is consistent with the findings in the present study. Since MPFLR does not improve patellar tilt or shift [21], the rationale for DFO could potentially be the reduction of the Q angle and medialisation of the patellar tendon insertion [35].

Several authors have stated that TT-TG distance >15 mm is a risk factor for RPD [36]. In one controlled laboratory study [37], patellofemoral kinematics and contact mechanics could not be restored with MPFLR if TT-TG distance >15 mm, and thus, TTM was recommended in this case. Clinically, TTM is recommended if TT-TG distance > 20 mm [38]. However, this realignment procedure remains controversial. According to the findings of Ostermeier et al. [39], TTM had no significant stabilising effect on patellar movement and relief of ligament loading. Matsushita et al. [40] demonstrated that TTM yielded similar clinical results regardless of TT-TG distance > 20 mm or not if isolated MPFLR was performed for RPD. Matsushita et al. deduced that TT-TG distance > 20 mm may not be an absolute operative indication for TTM. Thus, for the patients in the present study, correction of genu valgum deformity and MPFLR was considered necessary. None of the patients in the present study underwent TTM, but achieved satisfactory clinical and radiological results. Nha et al .[11] found that DFO could affect the TT-TG distance, which decreased from 20.4 mm preoperatively to 13.5 mm. Notably, no significant change was found in the TT-TG distance between before and after surgery, which could be attributed to the level of the osteotomy area in the present study. The osteotomies were all proximal to the femoral trochlea and therefore did not affect the relative relationship of the femoral trochlea and the tibial tubercle.

RPD with genu valgum in skeletally immature patients is also a common and difficult condition [41]. In the pediatric population, an in-depth understanding of the physes of the tibia and femur is critical when taking surgical treatment into consideration. Several studies have focused on guided growth techniques with or without MPFLR [1, 42, 43]. Parikh SN [43] found that, after about 1 year primary postoperation, genu valgum was corrected from 13.1° to 3.7° with satisfactory patellar by guided growth techniques combined with MPFLR. To address both instability and deformity, Avi Shah [42] established a technique of MPFLR in skeletally immature patients combined with hemiepiphysiodesis and revealed encouraging results without growth disturbances.

The present study has several limitations. First, this was a retrospective and non-comparative study, meaning that there were uncertainties over whether treating RPD due to valgus deformity by simple MPFLR, or MPFLR coupled with TTM would have sufficed. No articles were searched for the purposes of comparison, but as genu valgum is a significant risk factor for the failure of an isolated MPFLR [6, 7], while TTM can only correct the lateral malposition of the tibial tuberosity, and other anatomical predisposing factors theoretically remain. CWDFO combined with MPFLR may be regarded as advisable for RPD with genu valgum since there were significant improvements in clinical outcomes and radiological results as mentioned in several similar studies [10, 14, 23, 30, 31] and precaution against potential lateral tibiofemoral compartment osteoarthritis. Second, the present study had a relatively small sample size and short follow-up period. However, a minimum follow-up period of 2 years might be enough to demonstrate acceptable outcomes. Further investigations with a larger sample size and longer follow-up period are needed.

## Conclusion

Significant clinical and radiological results can be obtained in the treatment of RPD with genu valgum by CWDFO combined with MPFLR. During the 2-year follow-up period, all patients in the present study maintained the stability of the patellofemoral compartment, and none of the patients suffered redislocation. The clinical scores and radiological evaluations, except for the TT-TG distance and Insall index, exhibited significant improvement postoperatively.

## Data Availability

The datasets analyzed in this study are available from the corresponding author on reasonable request.

## Abbreviations

MPFLR: Medial patellofemoral ligament reconstruction
RPD: Recurrent patellar dislocation
CWDFO: Closing-wedge distal femoral osteotomy
TTM: Medialization of the tibial tuberosity
aFTA: Anatomical femorotibial angle
mLDFA: Mechanical lateral distal femoral angle
WBLR: Weight-bearing line rate
PLS: Patellar lateral shift
TT-TG: Tibial tubercle–trochlear groove
VAS: Visual analogue scale
